# GPS Swept Anti-Jamming Technique Based on Fast Orthogonal Search (FOS)

**DOI:** 10.3390/s21113706

**Published:** 2021-05-26

**Authors:** Mohamed Tamazin, Michael J. Korenberg, Haidy Elghamrawy, Aboelmagd Noureldin

**Affiliations:** 1 Electrical and Computer Engineering Department, Royal Military College of Canada, Kingston, ON K7K 7B4, Canada; haidy.elghamrawy@queensu.ca (H.E.); Aboelmagd.Noureldin@rmc.ca (A.N.); 2 Electronics and Communication Engineering Department, Arab Academy for Science, Technology and Maritime Transport, Alexandria P.O. Box 1029, Egypt; 3 Department of Electrical and Computer Engineering, Queen’s University, Kingston, ON K7L 3N6, Canada; korenber@queensu.ca; 4 Department of Engineering Mathematics and Physics, Faculty of Engineering, Cairo University, Giza 12613, Egypt

**Keywords:** GPS, fast orthogonal search, swept jamming

## Abstract

Recently, there has been growing demand for GPS-based reliable positioning, with the broadening of a range of new applications that mainly rely on GPS. GPS receivers have, recently, been attractive targets for jamming. GPS signals are received below the noise floor. Thus, they are vulnerable to interference and jamming. A jamming signal can potentially decrease the SNR, which results in disruption of GPS-based services. This paper aims to propose a reliable and accurate, swept anti-jamming technique based on high-resolution spectral analysis, utilizing the FOS method to provide an accurate spectral estimation of the GPS swept jamming signal. resulting in suppressing the jamming signal efficiently at the signal processing stages in the GPS receiver. Experiments in this research are conducted using the Spirent^TM^ GSS6700 simulation system to create a fully controlled environment to test and validate the developed method’s performance. The results demonstrated the proposed method’s capabilities to detect, estimate, and adequately suppress the GPS swept jamming signals. After the proposed anti-jamming module was employed, the software receiver was able to provide a continuous positioning solution during the presence of jamming within a 10 m positioning accuracy.

## 1. Introduction

During the past decade, autonomous systems have experienced continued growth across academia, industry (automotive, transportation, mining, oil, aviation, defense, etc.), military, and governmental agencies. Among these autonomous systems, self-driving cars, unmanned ground vehicles (UGVs), and unmanned aerial vehicles (UAVs) have drawn the most widespread attention.

Autonomous vehicles (AVs) typically rely on satellite-based navigation systems, such as a global positioning system (GPS), as the core positioning technology. However, GPS performance greatly suffers in degraded environments, such as signal blockage, multipath, and signal jamming. Many safety-critical applications will utilize AVs. Thus, it is crucial to protect such applications from any potential jamming or interference to avoid serious safety problems.

Jamming is considered a compelling threat to GPS service users [1,2] because it can badly affect the GPS receiver, causing unreliable and corrupted navigation solutions, loss of integrity, and satellite signal availability. The resultant disruption of the GPS service due to signal jamming could lead to serious consequences and risks. Loss of time synchronization, interruption of communication and network operations, and potential loss of life are examples of these consequences. Moreover, high-power jammers can disable GPS usage over a large area in the jammer’s vicinity.

FFT-based jamming detections and mitigation techniques are widely used in the literature [3,4,5]. These anti-jamming techniques use FFT to estimate the interfering source’s amplitude and frequency harmonics and remove them. The performance of such anti-jamming methods is limited by the FFT resolution, given by:

(1) FFT resolution=fsN

where $f_{s}$ is the sampling frequency and N is the number of data samples within the window size.

From Equation (1), as an example, the FFT resolution of data with a sampling rate $f_{s}=10\;\mathrm{MHz}$ and a window size of 1 ms is 1 kHz (simply the inverse of the window size). In this case, the FFT may have a low resolution to detect and estimate the frequency harmonics of some jamming signal types, such as swept GPS jamming. Even using an extended data window to improve the FFT method’s spectral resolution and consequently improving the FFT-based anti-jamming capability will result in higher computational complexity. Moreover, FFT requires an integral number of periods in the time series to model a frequency [6]. This results in potential inaccuracies in the spectral estimation of the swept jammer’s frequencies. Thus, the optimal choice of the window size and number of FFT points is essential for better identifying the swept jamming signal. This choice requires some knowledge of the jamming signal’s nature, which is unfeasible [7].

This paper’s primary objective is to develop a high-resolution spectral analysis technique based on fast orthogonal search (FOS). The jamming signal is modeled using a set of candidate functions and then suppressed from the received signal. Experiments in this research are conducted on GPS L1 signals using the GNSS Spirent^TM^ GSS6700 simulation system to create a fully controlled environment to test and assess the performance. The interference signal is obtained using an Agilent interference signal generator (ISG) connected to the Spirent^TM^ simulation system.

## 2. Proposed Method

This paper proposes a high-resolution pre-correlation swept anti-jamming technique based on Fast Orthogonal Search (FOS). The performance of the proposed technique is compared to the FFT-based method, and it shows better performance. This method provides robust spectral detection and estimation of the swept jamming signal with less computational load and complexity than FFT-based anti-jamming techniques for GPS receivers.

FOS [8] is a general-purpose non-linear modeling technique that has been used for system identification and time series analysis. FOS has also been used in spectral model estimation [8,9,10,11,12]. FOS is characterized by two important features that make it suitable for jamming mitigation. First, it has noise-resistance capabilities to both white and colored noise and it provides a high-frequency resolution that can reach FFTresolution/10 or finer resolution subject to signal-to-noise ratio (SNR). FOS will be used to model the interference signal identifying its magnitude, phase, and frequency; this is possible because the GPS signal is of very low power.

**Figure 1:**
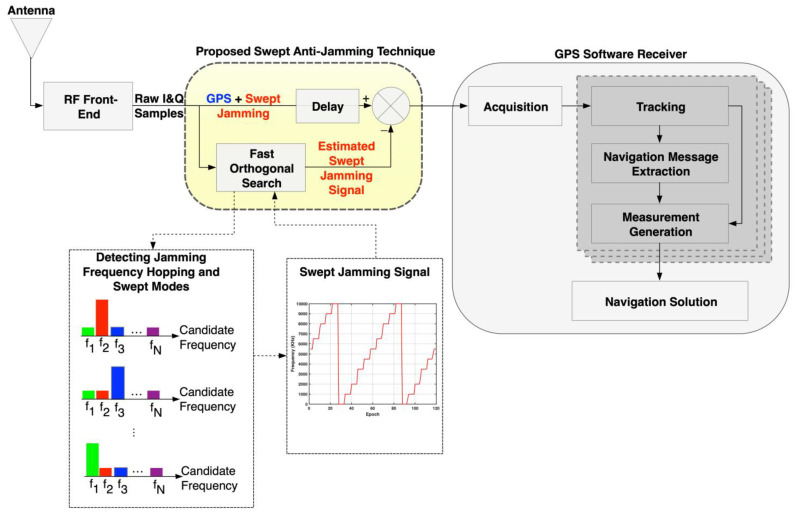
Structure of the proposed high-resolution pre-correlation anti-jamming technique.

The proposed method’s architecture is illustrated in Figure 1. The proposed method is located just after the front-end (FE) and before software processing begins, as seen in Figure 1. The proposed method’s input is the radio frequency (RF) front-end output, which is the raw in-phase/quadrature (I/Q) signal. The (I/Q) sampled data are the amplified, filtered and down-converted version of the received RF GPS signal.

In case a GPS signal contaminated with a swept jamming signal is received, the proposed FOS-based anti-jamming mitigation algorithm processes the (I/Q) signal. The proposed algorithm maintains a fixed window size of 1 ms (one epoch), which is the length of the GPS L1 C/A code. It operates epoch-wise to detect, estimate, and suppress the jamming signal epoch-by-epoch before entering the GPS receiver. Within each epoch, FOS is utilized to model the interference signal identifying its magnitude, frequency and phase and considering the useful GPS signal as white Gaussian noise (WGN).

As the jamming signal power level is much greater than the GPS signal power, the FOS algorithm can accurately detect the swept jamming signal characteristics, such as jamming frequency hopping and swept modes. Moreover, the proposed method will generate an estimated version of the swept jamming signal. The estimated jamming signal is then removed from the received signal by subtraction; then, the remaining signal, which is the GPS signal, is processed by the software receiver.

The FOS models an input signal y[n] as a linear combination of functions $p_{m}\left[n\right]$ selected from a set of arbitrary candidate functions. FOS aims to find the linear combination that minimizes the mean square error (MSE) fit to the input signal (i.e., the received signal).

(2) $$y\left[n\right]=\sum_{m=0}^{M}a_{m}\;p_{m}\left[n\right]+\varepsilon \left[n\right],$$

where $a_{m}$ are the functional expansion weights, m=0,1…M, and ε[n] is the modeling error. The $p_{m}\left[n\right]$ in (2) need not be orthogonal, which implies no unique solution for Equation (2); however, FOS can model a signal using fewer terms than an orthogonal functional expansion [12,13]. FOS carries out an implicit orthogonalization, while adding terms to the functional expansion. The algorithm begins by implicitly creating a functional expansion using orthogonal basis functions as given in Equation (3).

(3) $$y\left[n\right]=\sum_{m=0}^{M}g_{m}w_{m}\left[n\right]+\varepsilon \left[n\right],$$

where the $w_{m}\left[n\right]$ are mutually orthogonal functions derived from the candidate functions, $g_{m}$ are the weights, and ε[n] is the model error term. The orthogonal functions are obtained from the $p_{m}\left[n\right]$ using the Gram–Schmidt (GS) orthogonalization algorithm. The GS algorithm relates the orthogonal functions to the $p_{m}\left[n\right]$ through GS coefficients $\alpha_{mr}$ given by:

(4) $$\alpha_{mr}=\frac{\bar{p_{m}\left[n\right]w_{r}\left[n\right]}}{\bar{w_{r}^{2}\left[n\right]}},$$

The GS coefficients $\alpha_{mr}$ and the orthogonal weights $g_{m}$ can be found recursively using the following equations [8]:

(5) $$w_{0}\left[n\right]=p_{0}\left[n\right],$$

(6) $$D\left[m,0\right]=\bar{p_{m}\left[n\right]p_{0}\left[n\right]},$$

(7) $$D[m,r]=\bar{p_{m}\left[n\right]p_{r}\left[n\right]}-\overset{r-1}{\underset{i=0}{\sum }}\alpha_{ri}D[m,i],$$

(8) $$\alpha_{mr}=\frac{\bar{p_{m}\left[n\right]w_{r}\left[n\right]}}{\bar{w_{r}^{2}\left[n\right]}}=\frac{D\left[m,r\right]}{D\left[r,r\right]},$$

(9) $$C\left[0\right]=\bar{y\left[n\right]p_{0}\left[n\right]},$$

(10) $$C[m]=\bar{y\left[n\right]p_{m}\left[n\right]}-\overset{m-1}{\underset{i=0}{\sum }}\alpha_{mr}C[r],$$

and

(11) $$g_{m}=\frac{C\left[m\right]}{D\left[m,m\right]},$$

Accordingly, FOS skips the point-by-point computation of $w_{m}\left[n\right]$ as it is implicitly defined by the Gram-Schmidt coefficient $\alpha_{mr}$.

In its last stage, FOS recursively calculates the weights of the original functional expansion $a_{m}$ (Equation (2)), based on the weights of the orthogonal series expansion,$\;g_{m}$. The value of $a_{m}\;$can be found recursively using

(12) $$a_{m}=\overset{M}{\underset{i=m}{\sum }}g_{i}v_{i},v_{m}=1,$$

where,

(13) $$v_{i}=-\overset{i-1}{\underset{r=m}{\sum }}\alpha_{ir}v_{r},\;i=m+1,m+2,\;\ldots ,\;M,$$

From Equations (7),(8) and (10), it can be noted that FOS requires the calculation of time averages $\bar{p_{m}\left[n\right]p_{r}\left[n\right]}\;and\;\bar{y\left[n\right]p_{m}\left[n\right]}$. The time averages $\bar{y\left[n\right]p_{m}\left[n\right]}$ are typically calculated point-by-point, once at the start of the algorithm and then stored for later quick retrieval.

The MSE of the orthogonal function expansion is [8]:

(14) $$\bar{\varepsilon^{2}\left[n\right]}=\bar{y^{2}\left[n\right]}-\overset{M}{\underset{m=0}{\sum }}g_{m}^{2}\bar{w_{m}^{2}\left[n\right]}$$

It then follows that the MSE reduction given by adding the mth model term *p*_m_[*n*] is given by:

(15) $$Q_{m}=g_{m}^{2}\bar{w_{m}^{2}\left[n\right]}=g_{m}^{2}D\left[m,m\right],$$

FOS can fit a concise model by finding terms which reduce the mean squared error (MSE) in order of their significance. Moreover, FOS only accepts sinusoidal candidate terms which cause a sufficiently large MSE reduction that could only occur 2% of the time by chance. It is different from conventional methods, such as discrete Fourier transform (DFT), which may require many frequency components to represent the data. The FOS search algorithm is stopped in one of three cases. The first is when a maximum number of terms is fitted. The second case is when the ratio of MSE to the variance of the input signal is below a pre-defined threshold. The third case is when adding another term to the model reduces the MSE no more than expected from fitting white Gaussian noise (WGN).

Spectral analysis with FOS is accomplished by selecting candidates $p_{m}\left[n\right]$ that are pairs of sine and cosine terms at each of the frequencies of interest. The model terms are given by:

(16) $$p_{2m-1}\left[n\right]=\cos\left(2\pi f_{m}n\right),\;p_{2m}\left[n\right]=\sin\left(2\pi f_{m}n\right),\;m=1,2,\ldots$$

These model terms are selected from P candidate pairs where$\;\omega_{m}$ is the digital frequency of the candidate pair. By allowing a sine and cosine pair at each candidate frequency, the magnitude (Z) and phase (φ). at each selected candidate frequency (i.e., the jamming signal) can be determined using the following trigonometric identity:

(17) Xcos(2πfmfsn)+Ysin(2πfmfsn)=Zcos(2πfmfsn+φ),

where $Z=\sqrt{X^{2}+Y^{2}}$ and φ=tan−1(YX).

To choose the first frequency $\omega_{1}$ for the model, for each candidate frequency find the reduction Q(1) in mean squared error (MSE) if a sinusoid of that frequency alone were added to the model. From (16) with m=1, the sinusoid with frequency $\omega_{1}$ will have form *a*_1_*p*_1_[*n*] + *a*_2_*p*_2_[*n*]. When least square estimates are found for its coefficients $a_{1}$ and $a_{2}$, this candidate frequency will cause a MSE reduction of

(18) $$Q[1]=g_{1}^{2}\bar{w_{1}^{2}\left[n\right]}+g_{2}^{2}\bar{w_{2}^{2}\left[n\right]}=g_{1}^{2}D[1,1]+g_{2}^{2}D[2,2]$$

In [8], it is pointed out that an early rapid selection of model terms is possible for both system identification and time-series analysis. For example, when searching for the first sinusoidal frequency for the model, we evaluated Q[1] as a function of each candidate frequency. We can then use this function to select all model frequencies at once. Simply choose the candidate frequency with the largest Q[1] value and all candidate frequencies occurring at “relative maxima” of Q[1] that exceed a specified threshold level. This way, we need not wait for the algorithm to converge to final frequencies: we can make our choices for model frequencies directly from the plot of Q[1] as a function of each candidate frequency.

**Figure 2:**
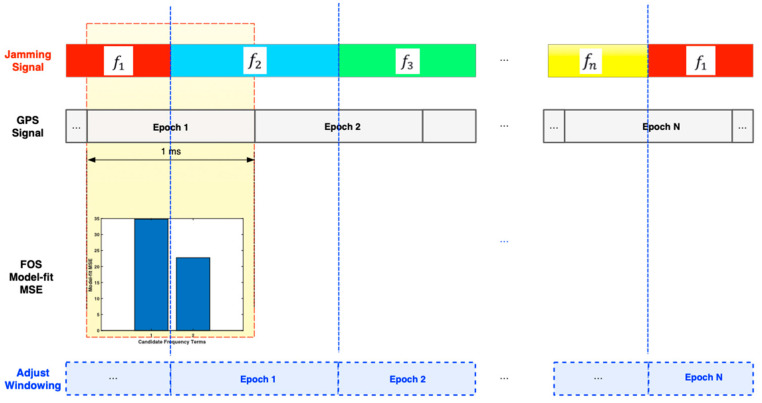
Adequate synchronization procedure using FOS.

There are at least two significant differences between FOS and conventional Fourier transform techniques (i.e., discrete Fourier transform (DFT) or FFT) [8,10,14,15]: (1) FOS produces a sinusoidal series representation that is more efficient and frugal (selects fewer components) by choosing the most significant sinusoidal components first; and (2) the frequencies of the selected sinusoids need not be commensurate nor integer multiples of the fundamental frequency corresponding to the record length [8]. Therefore, a better frequency resolution in the spectral model is achieved. Moreover, another advantage of the proposed method is that it can detect changes in the jamming signal frequency within the window size and apply an adequate synchronization procedure. Figure 2 shows the adequate synchronization procedure using FOS. The fine synchronization can be applied by examining the FOS model-fit MSE by comparing the MSE of the two FOS-found frequencies $f_{1,\;\;}\;and\;f_{2}$ within the half epoch. If at some point the MSE of the second candidate becomes higher than the first one, this can indicate a change in the jamming frequency in this epoch.

In this research, FOS is employed for swept jamming detection. As FOS is generally known to be a data-dependent algorithm, the FOS model’s accuracy depends on the data record being modeled, the candidate functions being used to compute correlations, and the stopping conditions (thresholds) in the algorithm. The chosen FOS candidate frequencies have a higher resolution than FFT to achieve better detection accuracy. Candidate frequencies can be selected so that the candidate functions focus on a particular frequency range of interest. For example, the candidates can be spaced with fine resolution over a particular range of interest, and outside this range, the coarser FFT resolution intervals can separate the candidates.

It is desirable to have the minimum number of candidate frequencies in the spectral estimate, representing the received signal’s most significant components. However, creating a model that incorporates fewer than necessary terms will result in an inaccurate representation of the received signal. On the other hand, the excessive inclusion of terms will add noise terms into the received signal’s spectral estimate and increase the computation time.

FOS stops modeling when adding a new frequency pair does not increase the MSE reduction more than the reduction expected from fitting white Gaussian noise (WGN). Thus, a candidate acceptance threshold, requiring a frequency pair to provide a minimum percentage of the signal’s overall energy, is set. Such a threshold allows FOS to reject frequency terms that merely model the noise.

## 3. Experimental Setup

**Figure 3:**
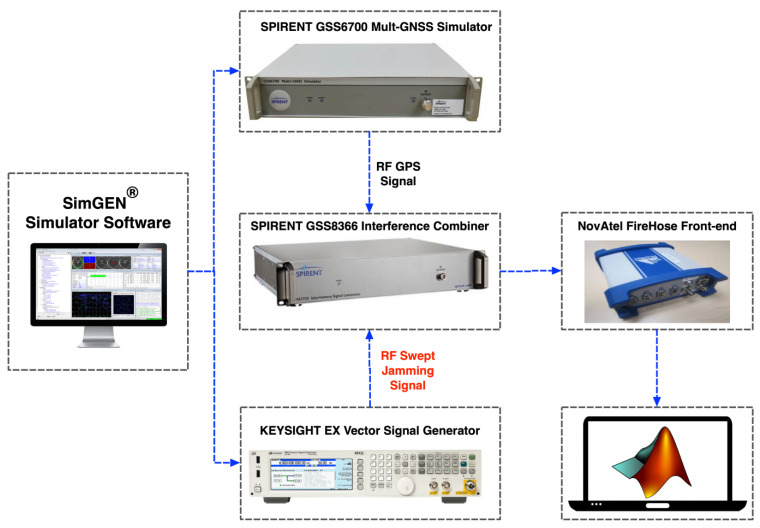
Block diagram of the experimental setup.

To evaluate and validate the proposed method’s performance, several realistic simulation scenarios have been conducted in the Navigation and Instrumentation research laboratory at the Royal Military College of Canada (RMCC) using GNSS Spirent^TM^ GSS6700 simulation system [16]. The simulator is utilized in this research to create a fully controlled testing environment. Figure 3 demonstrates the block diagram of the experimental setup. The Spirent simulation system is operated using SimGEN^®^ software [17]. The SimGEN^®^, in general, enables the simulation of ionospheric and tropospheric degradation of GNSS signals, terrain obscuration, several multipath effects, variable antenna reception gain and phase patterns, trajectory generation of air, sea, land and space vehicles, and comprehensive error generation.

As shown in Figure 3, firstly, the simulation scenario is created using the SimGEN^TM^ simulation software. The Spirent^TM^ GSS6700 simulator accordingly outputs an RF GPS signal. Simultaneously, the KEYSIGHT EXG vector signal generator (N5172B) generates the swept interference signal. Then, the SPIRENT GSS8366 interference combiner combines both the output RF GPS signal from Spirent^TM^ GSS6700 with the swept RF jamming signal generated from the KEYSIGHT signal generator. The output of the RF signals is connected to the NovAtel FireHose D17088 [18] front-end. The FireHose front-end down-converts the received signal from RF to baseband through a number of down-conversion stages generating I and Q signals. Finally, the raw I and Q signals collected by FireHose are stored and post-processed by the NavINST research group software receiver.

**Figure 4:**
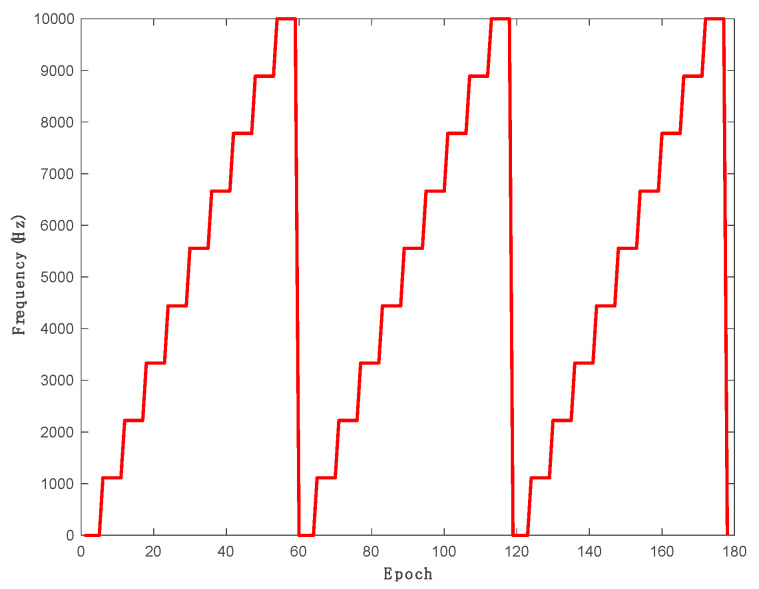
Step linear swept jamming signal.

The swept jamming signal is used for the experiments presented in this work, and it was generated using the KEYSIGHT EXG vector signal generator (N5172B). Table 1 summarizes the swept jamming signal parameters, respectively. Figure 4 demonstrates the step linear swept jamming signal used for this research.

## 4. Results and Analysis

**Figure 5:**
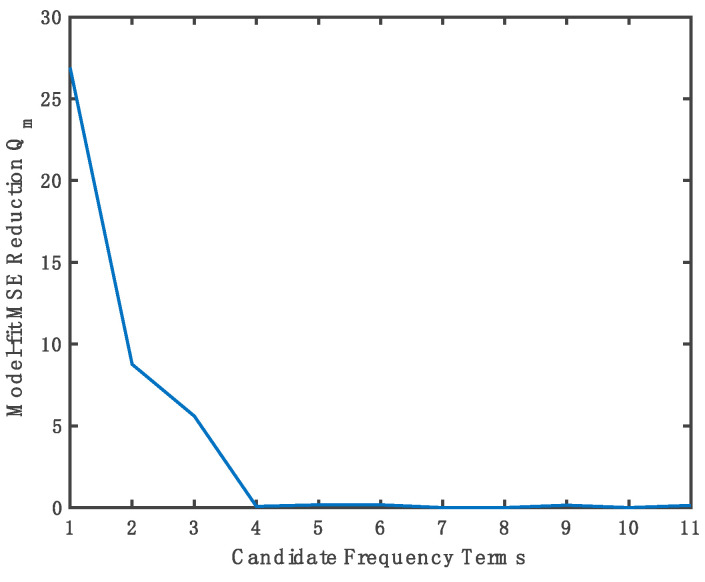
Model-fit MSE reduction Q_m_ versus the number of candidate terms of modeling the measured correlation function of GPS satellite PRN 10.

The performance of the proposed GPS Swept FOS-based anti-jamming technique was initially compared with the conventional FFT-based anti-jamming technique algorithm in which the radix-2 FFT algorithm was used [4,11,19]. The radix-2 FFT can only be performed with sequences of $2^{n}$ data length. Unfortunately, most sampling rates do not provide the required power-of-two data length. Therefore, the vector of signal samples is extended by using zero-padding to a length of L samples, where $L=2^{n}$ is a power-of-two data size with a frequency resolution of fsL Hz. Moreover, the proposed method is compared to zero-padding FFT-based Anti-jamming algorithms [4,20]. These algorithms are based on adding power-of-two zero-padding blocks to the data samples by finding the next highest power-of-two of the data length (i.e., $n=log_{2}\;L$) and multiplying it by a factor of i, where i=2, 3, 4,…etc. To apply the zero-padding FFT-based Anti-jamming with a factor of 2 (namely FFT-ZP (2 × *n*)) the total data length will be $L=2^{i\times n}$. Thus, the proposed method has been evaluated and compared with FFT-based and (FFT-ZP)-based anti-jamming in the three main modules of the software receiver, namely pre-correlation, acquisition, and tracking modules. As shown in Figure 5, the proposed method successfully modeled the received jamming signal using 11 pre-defined candidate functions. Remarkably, none of the remaining candidates can yield a sufficient MSE reduction value.

**Figure 6:**
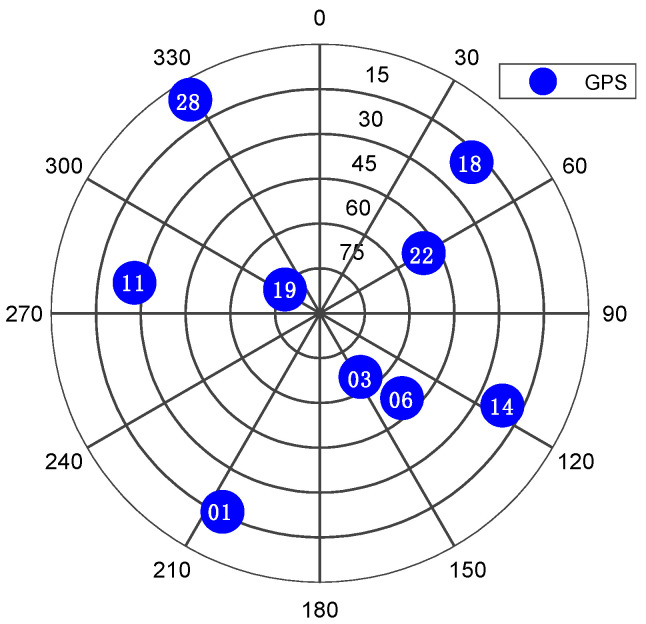
Skyplot of the GPS satellites in view during the experiment.

**Figure 7:**
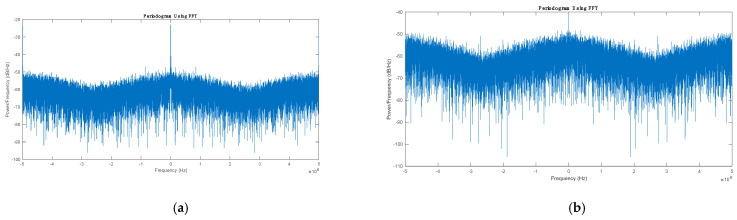
Power spectral density. (**a**) Before applying the proposed method; (**b**) after applying the proposed method.

To verify the proposed method’s performance, real GPS L1 data were generated using the Spirent GSS6700 simulator and logged using NovAtel FireHose front-end. The tests were performed in static mode. The starting point was chosen at a point in Kingston, Ontario, at latitude 44°13.726′, longitude −76°27.948′ and height 100 m. There are nine GPS satellites available above a 5-degree elevation mask at the initial location, as shown in Figure 6. The real RF data were down-converted to the baseband and sampled at a frequency of 10 MHz and quantified with 4 bits. The raw GPS samples were processed using NavINST research group software receiver. To verify that the jamming signal was successfully suppressed, the time series of the signal is converted to the frequency domain, as shown in Figure 7. It is clear from the figure that the jamming signal peak was highly attenuated after applying the proposed anti-jamming algorithm.

**Figure 8:**
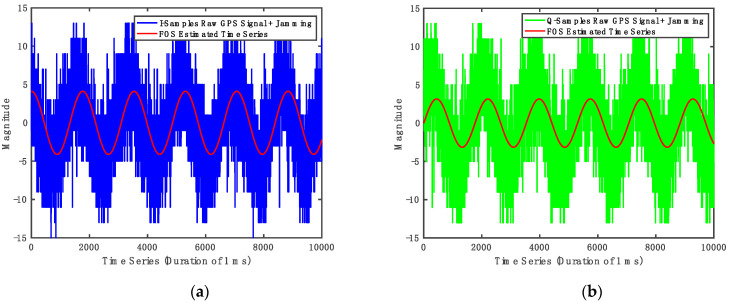
FOS Estimated Jamming Signal. (**a**) In-phase (I) time-series signal; (**b**) quadrature (Q) time series signal.

Figure 8 illustrates 1 ms of the raw in-phase (I) and quadrature (Q) GPS time-series signal and the FOS estimated time-series signals. The sinusoidal jamming signal dominates the received signal in the presence of jamming. In other words, the received signal looks like a sinusoidal signal with some additive noise. Figure 8 shows that the proposed method was successfully able to synchronize the received signal with FOS estimated jamming time-series signal. 

### 4.1. Proposed Method Accuracy

**Figure 9:**
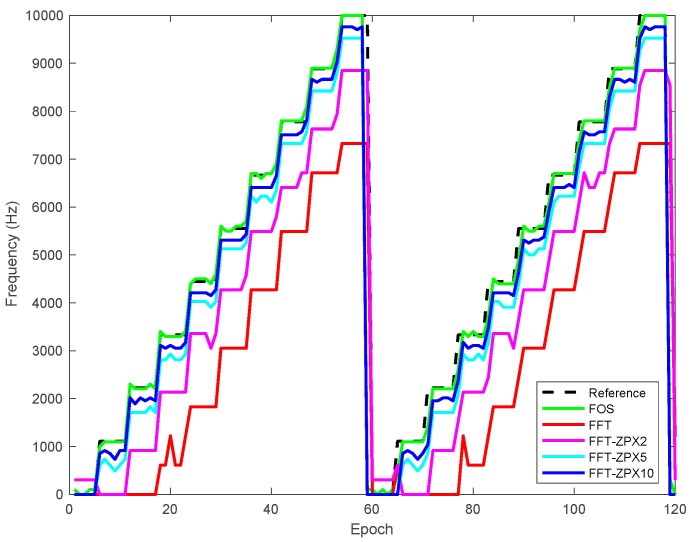
Estimated swept jamming signal frequency using the proposed method, FFT, and zero-padding FFT-based anti-jamming algorithms.

Figure 9 demonstrates the proposed method’s performance, FFT and zero-padding FFT-based anti-jamming techniques in detecting and estimating the swept jamming signal frequencies. (FFT-ZPX2), (FFT-ZPX5), and (FFT-ZPX10) refer to using the zero-padding FFT-based anti-jamming technique at factors *i* = 2, 5 and 10, respectively. As shown in Figure 9, the proposed method outperforms the other methods in estimating the swept jamming signal frequencies.

**Figure 10:**
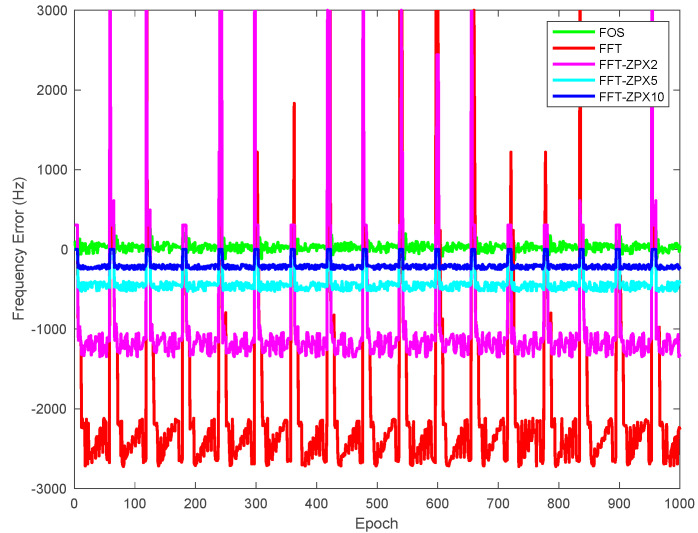
Estimated swept jamming signal frequency errors using the proposed method, FFT, and zero-padding FFT-based anti-jamming algorithms.

Figure 10 shows the estimated frequency error of the proposed method compared to FFT and zero-padding FFT-based anti-jamming techniques. The frequency error values of the estimated jamming signal produced by the proposed method are considerably smaller than those produced by the other methods. This shows the capabilities of the proposed method to accurately estimate the swept jamming signal frequencies, which leads to mitigating it before entering the signal processing modules of the GPS receiver.

**Figure 11:**
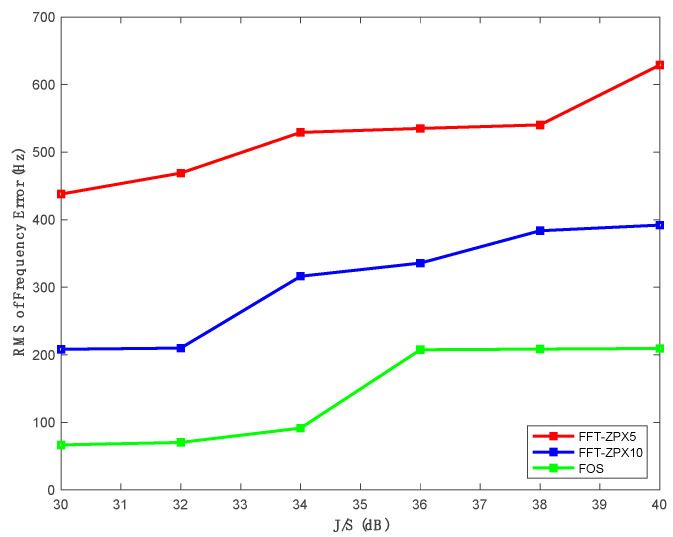
RMS of frequency errors vs. different J/S using the proposed method and the other two methods.

To examine the proposed algorithm’s performance against different J/S ratios, the IF GPS data with J/S from 30 to 40 dB were generated using the Spirent simulator. Figure 11 compares the proposed method’s performance, zero-padding FFT-based anti-jamming techniques in estimating the swept jamming frequencies under different J/S ratios. The RMS values of the swept jamming frequency errors computed by the proposed method are smaller than those produced by the other methods for the different J/S ratios.

### 4.2. Acquisition Results

**Figure 12:**
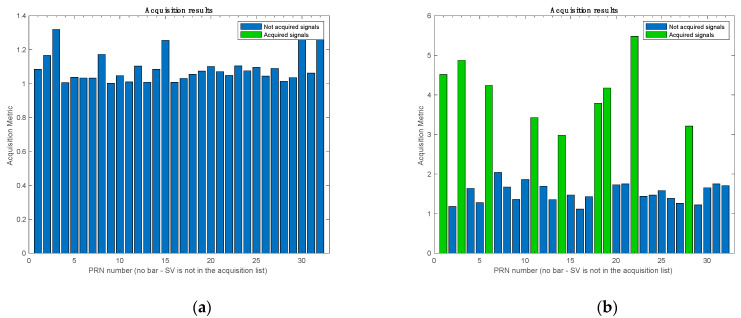
Acquisition results (**a**) before applying the proposed method and (**b**) after using the proposed method.

Figure 12 shows the acquired GPS satellites based on the acquisition metric, which is defined as the ratio between the first highest peak and the second highest peak in the acquisition search space. Typically, a specific threshold is chosen for the acquisition metric, and the satellites that pass this threshold are acquired. As depicted in Figure 12a, the software receiver failed to acquire any satellite. The failure to acquire at least four satellites means that the receiver was not able to compute a navigation solution. On the contrary, after the anti-jamming module was employed, the software receiver was able to acquire all nine visible satellites, as shown in Figure 12b. Table 2 lists the experiment’s acquisition results.

### 4.3. Tracking Results

**Figure 13:**
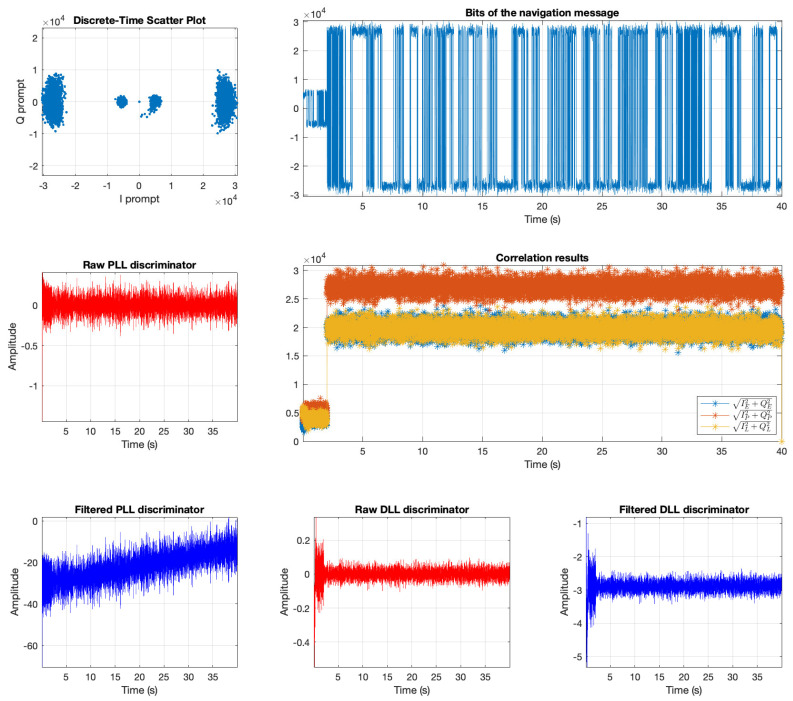
Tracking results of PRN 19.

The software receiver without the anti-jamming module failed to track the satellites or relock to the signals during the swept jamming period. However, the software receiver’s tracking module with the proposed anti-jamming technique could track the code phase and Doppler shift of all satellites during the presence of jamming except for only a few seconds at the start of the jamming interval. Figure 13 shows the tracking results of the satellite PRN 19. As a result of the software, the receiver successfully tracked all signal parameters of the received signal. This satellite’s navigation message was decoded, which will be used to extract the ephemeris information. 

**Figure 14:**
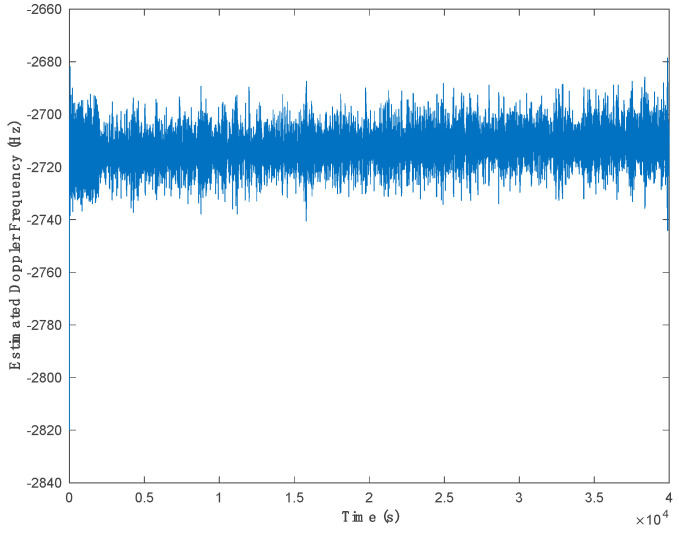
Estimated Doppler frequency of PRN 19.

Figure 14 shows the estimated Doppler shift for PRN 19. Since it is difficult to acquire the real Doppler shift in real experiments, the results of frequency lock loop (FLL) are provided, and the frequency lock indicator (FLI) was used as a performance metric to evaluate the proposed method compared to the other methods. Generally, the FLI can be used to assess the performance of frequency tracking. FLI [4,21] is a function of frequency error and integration time and is given as:

(19) $$FLI=\cos\left(4\pi \delta fN_{C}\right),$$

where δf is the frequency error in the tracking loop and $N_{C}$ is the integration time. For example, for a 10 ms integration time, FLI = 0.9 means that the frequency tracking loop frequency error is 3.6 Hz.

**Figure 15:**
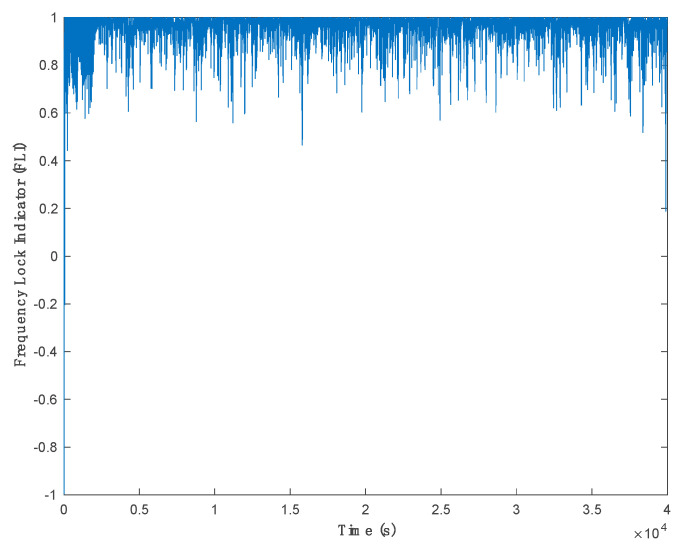
Frequency lock indicator calculated for PRN 19.

A good carrier frequency tracking performance results in a reliable extraction of the navigation data bits. The criteria for assessing the proposed method in the real experiment in this research is based on the frequency tracking performance indicated by the FLI and the conversing process of the FLL to lock the correct satellite Doppler shift. Figure 15 depicts the converging processes of FLL for PRN 19.

### 4.4. Navigation Solution Results

**Figure 16:**
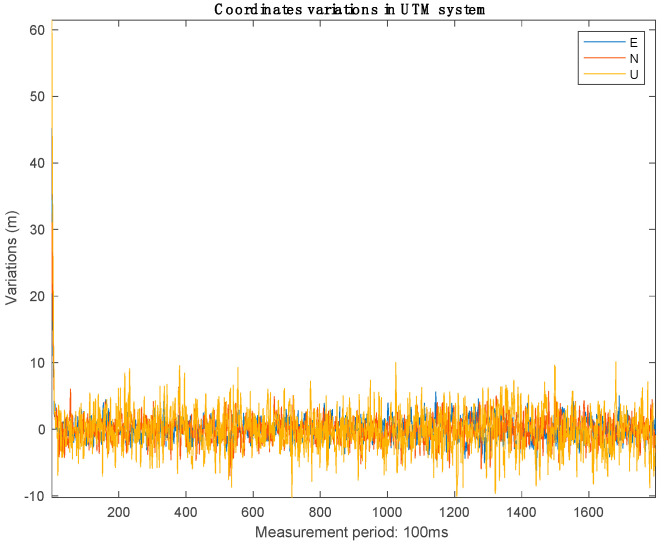
East, north, and up position errors.

**Figure 17:**
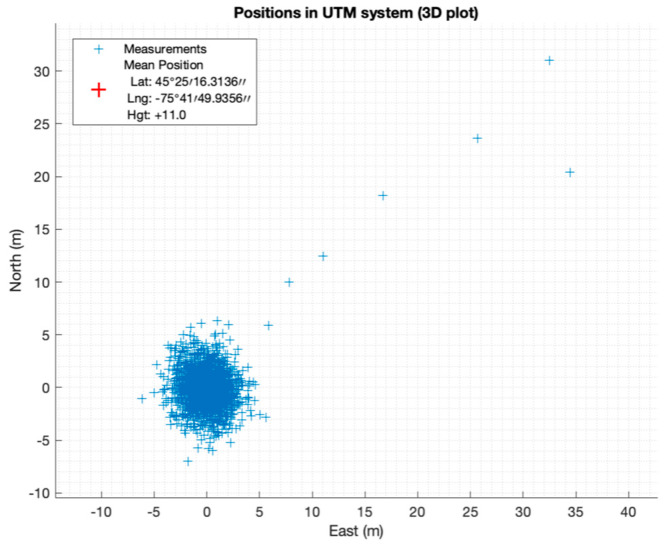
Horizontal Position Error.

Since the software receiver without the proposed anti-jamming technique could not acquire and track the received signals from the available satellites, it shows a navigation outage during the jamming period. Figure 16 and Figure 17 show the position error calculated using the software receiver with the proposed anti-jamming technique. By examining the figures, the software provides a continuous positioning solution during the presence of jamming, and the navigation solution is still acceptable with 10 m accuracy. 

## 5. Conclusions

This paper proposes a high-resolution spectral analysis utilizing fast orthogonal search (FOS) to estimate the swept jamming signal. The proposed method’s main advantage is that it is decoupled from the receiver and does not require any modification in the receiver structure, and it never requires knowing future values of the acquired signal. The proposed anti-jamming modules’ performance was assessed in the acquisition, tracking, and navigation stages within a GPS software receiver. After applying the jamming mitigation techniques, the acquisition peak emerged from the noise floor and was easily identifiable. The tracking and navigation results show that the proposed technique was efficient in accurately detecting and canceling the swept jamming signal, which was verified by the use of GPS software-defined receiver components that could operate reliably despite the presence of the jamming signal. The proposed algorithm is suitable for real-time processing. As future work, the augmentation of the proposed anti-jamming scheme with other RF detection and mitigation techniques will give more attention to multi-antenna techniques.

## Figures and Tables

**Table 1:** Jamming signal parameters.

Item	Description
Jamming Type	Sweep Frequency
Sweep Type	Step Linear
Frequency Start	1.57542 GHz
Frequency Stop	1.57543 GHz
Frequency Step Size	1.11111 kHz
Step Points	10
Step Dwell	6 ms
Sweep Speed	187.52 pps

**Table 2:** Summary of the acquisition results.

Channel	PRN	Frequency	Doppler	Code Offset
1	19	−7.91550 × 10^2^	−792	6903
2	3	1.67847 × 10^3^	1678	9380
3	1	−3.71933 × 10^3^	−3719	8936
4	22	1.93596 × 10^3^	1936	3749
5	6	2.37465 × 10^3^	2375	2479
6	18	3.33786 × 10^3^	3338	7987
7	28	−2.85149 × 10^3^	−2851	4022
8	14	−2.56538 × 10^3^	−2565	2180
9	11	−2.70844 × 10^3^	−2708	5814
